# Performance of ^18^F-sodium fluoride positron emission tomography with computed tomography to assess inflammatory and structural sacroiliitis on magnetic resonance imaging and computed tomography, respectively, in axial spondyloarthritis

**DOI:** 10.1186/s13075-019-1903-1

**Published:** 2019-05-14

**Authors:** Marie Raynal, Fehd Bouderraoui, Remy Ouichka, Julian Melchior, Olivier Morel, Alain Blum, Isabelle Chary-Valckenaere, Willy Ngueyon Sime, Véronique Roch, Walter Maksymowych, Robert G. Lambert, Pierre Olivier, Damien Loeuille

**Affiliations:** 1 Department of Rheumatology, CHRU Nancy, 5 Rue du Morvan, 54500 Vandœuvre-lès-Nancy, France; 2 Department of Nuclear Medicine, CHRU Nancy, Vandœuvre-lès-Nancy, France; 3 Department of Radiology, CHRU Nancy, Vandœuvre-lès-Nancy, France; 4 Department of Epidemiology, CIC 1433, CHRU Nancy, Vandœuvre-lès-Nancy, France; 5 Canadian Research Education (CaRE) Arthritis, Edmonton, Canada; 6 Department of Radiology, University of Alberta, Edmonton, Canada

**Keywords:** Spondyloarthritis, Sacroiliitis, Positron emission tomography, Inflammation

## Abstract

**Purpose:**

To assess increased sacroiliac joint (SIJ) uptake on ^18^F-NaF PET/CT and to compare with MRI for inflammation and with CT scan for structural damages in a population of 23 patients with spondyloarthritis (SpA).

**Methods:**

Twenty-three patients with active SpA according to the Assessment of SpondyloArthritis international Society (ASAS) and/or modified NY criteria were included. All patients had a pelvic radiograph, MRI, and CT scan of the SIJ and ^18^F-NaF PET/CT examinations within a month, analyzed by three blinded readers. MRIs were assessed according to the ASAS criteria and SPARCC method. On CT scans, erosion and ankylosis were quantified using the same methodology. On the ^18^F-NaF PET, abnormal uptake was assessed using a qualitative method inspired by the ASAS criteria and two quantitative approaches (the PET-activity score according to the SPARCC method and Maximum Standardized Uptake Value (SUVmax)).

**Results:**

Structural sacroiliitis was observed on 7 radiographs and 10 CT scans; 10 MRIs showed inflammatory sacroiliitis, and 20 patients had a positive PET. The inter-reader reliability was good for the PET activity score and good to excellent for the SUVmax. A positive PET was not correlated with a positive MRI or with a structural sacroiliitis on CT scan. The PET-activity score and SUVmax were correlated with the SPARCC inflammation score but not with erosion or ankylosis scores on CT scan.

**Conclusion:**

Abnormal uptake by the SIJ on ^18^F-NaF PET is more frequent than inflammatory and structural sacroiliitis in a population of SpA patients. The PET activity score and SUVmax had good correlations with inflammatory sacroiliitis but not with structural lesions on CT scan.

## Introduction

Spondyloarthritis (SpA) is a group of chronic inflammatory rheumatic diseases that mainly affects the axial skeleton as well as the peripheral joints and enthesis [1–3]. The modified New York criteria define ankylosing spondylitis (AS) as the association of clinical criteria with radiological sacroiliitis (at least grade 2 bilateral or grade 3 unilateral) [4]. Since 2009, diagnosis of axial SpA has been based on the Assessment of SpondyloArthritis international Society (ASAS) classification criteria and can be made without radiological sacroiliitis, if sacroiliitis is detected on MRI, corresponding to the presence of bone marrow edema on STIR sequences [5, 6]. This inflammation can be detected before structural changes, which allows for an earlier diagnosis of axial SpA. Furthermore, it has been shown that syndesmophytes seem to preferentially develop at vertebral corners where both fatty lesions and bone marrow edema are present [7–9]. In fact, according to different studies, 57.4 to 94% of syndesmophytes develop in vertebral units without active inflammation [10–12]. Since the pathophysiological mechanisms leading to the syndesmophyte formation are not clearly identified on MRI, it appears relevant to evaluate new imaging techniques dedicated to bone remodeling. The diagnostic potential of nuclear imaging with positron emission tomography (PET) has been investigated in several inflammatory diseases, such as polymyalgia rheumatica, vasculitis, rheumatoid arthritis, and SpA [13, 14]. The relevance of PET imaging depends on the radiotracer used, and in axial SpA, Bruijnen and colleagues showed that the activity is better reflected by bone formation (using the ^18^F-fluorid radiotracer, which shows osteoblastic activity) rather than inflammation (with ^18^F-FDG and ^11^C-PK11195, showing inflammation by glucose metabolism and neutrophil recruitment) [15, 16]. In fact, recent studies on ^18^F-fluoride PET have shown the potential for this technique to be used in the diagnosis of sacroiliitis [17, 18]. In the SIJ, the ^18^F-fluoride uptake is moderately associated with inflammatory lesions but not with structural damage on MRI [19] in a small sample of AS patients. The association between radiotracer uptake and the structural lesions on CT scan has not yet been evaluated.

The aim of our study was to compare increased sacroiliac joint (SIJ) uptake on ^18^F-fluoride sodium (^18^F-NaF) PET combined with CT scan with structural damages on CT scan according to a qualitative and quantitative approach in patients with axial SpA. We also evaluated the correlation between increased uptake of the SIJ on ^18^F-NaF PET/CT and the presence of inflammatory lesions on MRI.

## Patients and methods

### Patients

This single-center prospective study was conducted on 23 patients with axial or mixed SpA who were between the ages of 18 and 45 when the diagnosis was made, according to the ASAS or modified New York criteria. These patients were recruited at our institute between January 2013 and October 2014.

The inclusion criteria were the following: active SpA (Bath Ankylosing Spondylitis Disease Activity Index (BASDAI) ≥ 4 and/or in NSAID treatment failure) and with inflammation in the spine (at least three inflammatory vertebral corners) and/or the SIJ (inflammatory sacroiliitis according to ASAS criteria). After providing information, the patients were included and provided informed consent (IDRCB: 2012-A00568-35; ClinicalTrials.gov: NCT 02869100). ^18^F-NaF PET/CT, CT scans, and MRI were performed within a month. In order not to interfere with the results of ^18^F-NaF PET, the treatment could not be changed until it was complete.

The exclusion criteria as related to the realization of PET/CT were the following: a confirmed or suspected ongoing pregnancy or breastfeeding, kidney failure with creatinine clearance under 60 mL/min, previous or current chronic alcoholism or drug addiction, psychiatric disease, severe comorbidities, and a legal protection measure.

The following data were recorded: age, tobacco use, familial history, disease duration, extra-articular involvement, treatment, BASDAI, Bath Ankylosing Spondylitis Functional Index (BASFI), Bath Ankylosing Spondylitis Metrology Index (BASMI), Ankylosing Spondylitis Disease Activity Score (ASDAS), and biologic parameters (HLA-B27, C-reactive protein (CRP), erythrocyte sedimentation rate (ESR), creatinine, and creatinine clearance).

All patients underwent a conventional pelvic radiograph, MRI, and CT scan dedicated to the SIJ along with a^18^F-NaF PET/CT within a month.

This study was approved by the French ethic committee (“comité de protection des personnes”: CPP number 12.06.03).

### Imaging and scoring

#### Conventional radiography

Anteroposterior radiography of the pelvis was completed and analyzed according to the New York modified criteria by one rheumatologist who defined the presence or absence of sacroiliitis.

#### MRI

The MRI centered on the SIJ was completed on a 3T MRI machine (SignaHDxT MR 750 W, GE Healthcare) with a matrix of 416 × 320. The images were reconstructed in the semicoronal plane parallel to the superior border of the sacrum with T1-weighted (TR 400 to 600 ms; TE < 20 ms, ETL 3) and T2-weighted sequences with fat suppression (TR 3000 ms, TE > 65 ms, ETL 28). The slice thickness was 3.5 mm with a gap of 0.5 mm. The SIJ exam was performed on approximately 20 slices for a complete exploration of the SIJ.

#### CT scans

Dedicated SIJ CT scans were conducted the same day on a TOSHIBA Aquillion One imager. The acquisition parameters were as follows: field of view, 12 cm; acquisition matrix size, 512 × 512 pixels; tube voltage 120–130 kV; tube current, 200 mA; rotation time, 0.75 s; axial slice thickness, 0.5 mm; and interslice gap, 0.25 mm. Morphological assessment of the SIJ was performed on 30 semi-coronal reconstructions without gap with a slice thickness of 1.5 mm and a bone filter.

#### ^18^F-NaF PET/CT

The examination was started 60 min after direct intravenous injection of 4 MBq/kg of ^18^F-NaF using a hybrid imaging PET/CT Biograph 6 (SIEMENS, Knoxville, TN). First, a scan was performed using a true whole body field of view without contrast agent (intensity 130 kV for 80 mAs, 0.6 s of tube rotation time, cuts of 3 mm, and pitch of 1.5). Second, the PET acquisition was also performed with a true whole body field of view with 9–12 bed positions for a complete examination duration of 20 to 30 min. The image reconstruction was done using an iterative method (3 iterations, 8 subsets, 168 × 168 matrix with zoom 1, Gaussian filter, and 5.0 mm FWHM) before being displayed on a Leonardo® workstation (SIEMENS, Knoxville, TN). PET analysis of the SIJ was done using a slice thickness of 5 mm.

### Scoring method

Conventional radiographies were analyzed according to the modified New York criteria to define structural sacroiliitis (at least bilateral grade 2 or unilateral grade 3).

#### CT scans

First, diagnosis of structural sacroiliits was performed based on the presence of erosion (interruption of the sacral or iliac cortical bone present on at least two consecutive slices) and/or ankylosis (partial or complete bone bridge present on at least two consecutive slices) on the cartilaginous part of the SIJ.

**Fig. 1:**
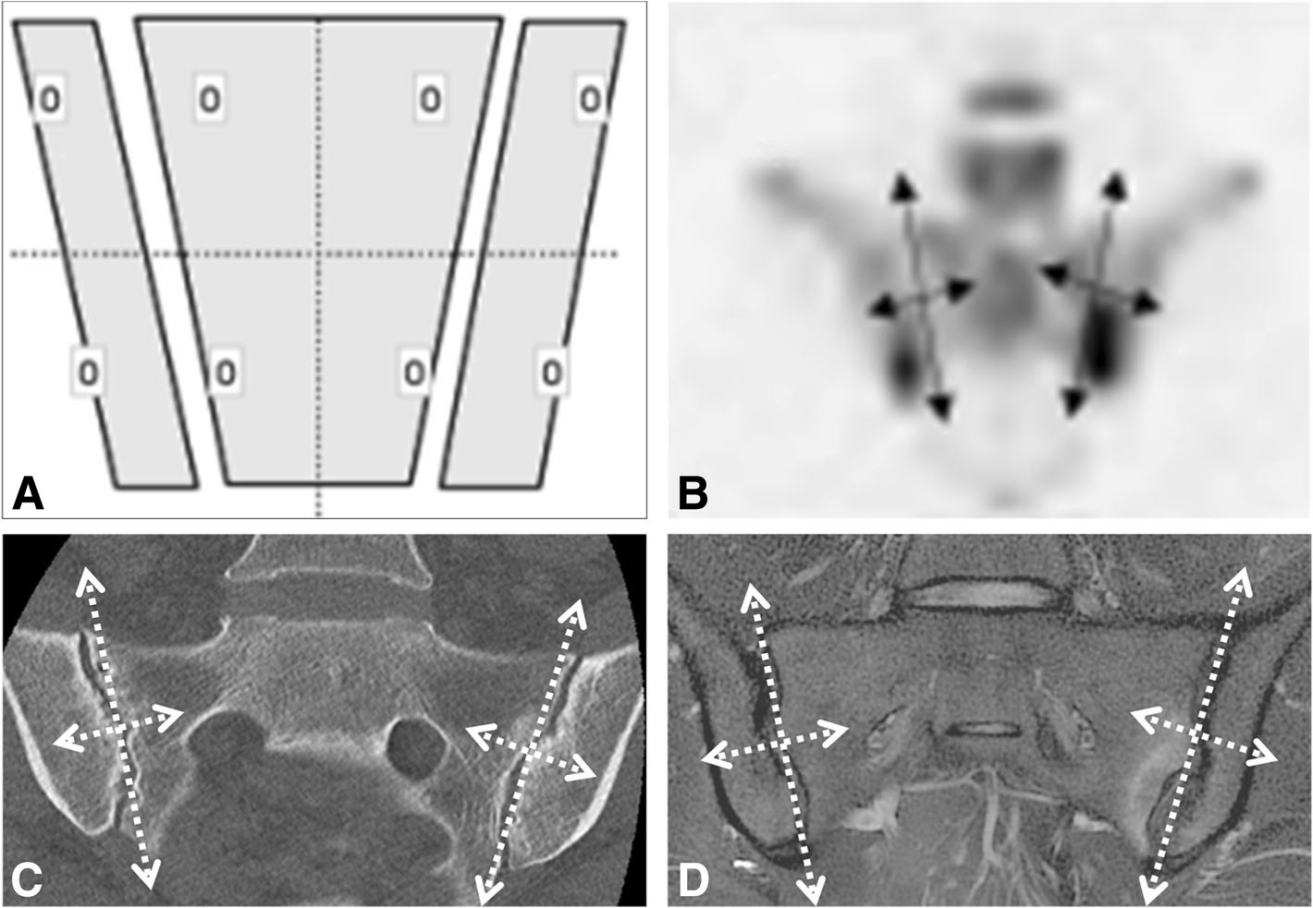
Methodology of the semi-quantitative evaluation of the SIJ using for quadrants, based on the SPARCC MRI SIJ inflammation score on the cartilaginous part of the SIJ (**a**). Application on PET/CT (**b**), CT scan (**c**), and MRI (**d**)

Second, a score for structural lesions (as defined above) was established using a methodology similar to that described in the SpA Research Consortium of Canada (SPARCC) MRI SIJ inflammation score on the cartilaginous part of the SIJ [20] (Fig. 1). The most anterior slice was defined as a visible joint ≥ 1 cm in vertical height. When the vertical height was less than 3 cm, the SIJ was defined as having only 2 quadrants (upper iliac and upper sacrum), whereas a visible joint ≥ 3 cm in vertical height was defined as having 4 quadrants (upper iliac, lower iliac, upper sacrum, and lower sacrum). At the posterior aspect of the SIJ, each quadrant was assessed individually until < 1 cm of vertical height was visible when it was no longer scored. Each SIJ was divided into these four quadrants for erosions or into two halves for ankylosis (upper and lower). The readers scored the lesions on all of the quadrants for each slice on a dichotomous basis (present/absent) with the slice scores for erosion and ankylosis varying from 0 to 8 and 0 to 4, respectively. The final score was the sum of the scores for all of the slices, with a maximum of 20 slices (the final score for erosions and ankylosis ranged from 0 to 160 and from 0 to 80, respectively).

#### MRI

First, the presence of inflammation was assessed on a binary approach according to ASAS criteria by defining active sacroiliitis as subchondral or periarticular bone marrow edema, present on more than one lesion, even if in a single section, or present on at least two sections if there was only one lesion.

Second, inflammation was scored according to the SPARCC MRI SIJ inflammation score [20] in consecutive slices on a dichotomous basis (present/absent) on the entire cartilaginous part of the SIJ, using the same methodology of 2 or 4 quadrants depending on the size of the joint (Fig. 1). The final score for inflammation was the sum of the scores for all of the slices, with a maximum of 8 slices, and ranged from 0 to 64.

For both CT scans and MRI, the presence of structural lesions and inflammation was retained if it was scored by at least two readers, and the mean score among the three readers was calculated. Both the MRI and CT scans were scored independently by two rheumatologists and one radiologist blindly on a website (carearthritis.com) after anonymization and randomization.

Before starting the lectures, the three readers completed a calibration exercise on a population of 46 SpA patients with paired MRI and CT exams using the same methodology. The results of this calibration showed good concordance for global diagnosis of structural sacroiliitis on CT scan (ICC[IC95] = 0.65–0.74[0.37; 1]) and for the presence of erosions (ICC[IC95] = 0.65–0.82[0.34; 1]) and ankyloses (ICC[IC95] = 0.55–0.88[0.16; 1]). On MRI, a calibration exercise obtained good concordance (ICC[IC95] = 0.62–0.72[0.29; 1]) for the diagnosis of inflammatory sacroiliitis.

#### ^18^F-NaF PET/CT

The analysis was blinded from clinical data, MRI, and CT scans. The SIJ assessment was made by three readers. The signal was considered abnormal if it was higher than the signal in the center of the sacrum (S2).

First, a qualitative assessment was conducted on the articular part of the SIJ based on an adaptation from the ASAS criteria for MRI. The exam was considered positive if there was unilateral uptake on two consecutives slices or bilateral uptake on one slice.

Second, a quantitative assessment was conducted using two methods:

1. The PET activity score was calculated based on the SPARCC MRI SIJ inflammation score method: cartilaginous part of the SIJ was divided into the same 2 or 4 quadrants depending on the size of the joint, and the abnormal uptake was scored in each quadrant for each slice on a dichotomous basis (present/absent) (Fig. 1). The final score was the sum of the scores for all slices, with a maximum 6 slices, and ranged from 0 to 48.
2. The maximum standardized uptake value (SUVmax) was measured slice-by-slice for each SIJ on a predefined circular region of interest, and the highest SUVmax value was considered for each SIJ. The ratio between the SUVmax for each SIJ and the SUVmax in the center of the sacrum (S2) was calculated (SUVmax SIJ/sacrum).

As for CT scans and MRIs, the presence of increased uptake was retained if it was scored by at least two readers, and the mean score among the three readers was calculated for the quantitative assessments.

### Statistical analysis

The intensity or quality of the agreement between the inflammatory sites in MRI and ^18^F-NaF PET uptake was done by Kappa concordance coefficients. To compare qualitative variables, Fisher’s test was carried out, and for quantitative variables, Student’s *t* test was used, as the data were normally distributed. Statistical analysis was performed using SAS 9.3 software.

The same statistical analysis was performed between the CT scans and PET data.

## Results

The characteristics of the population are detailed in Table 1.

**Table 1:** Characteristics of the population

	*N* (%)	Median	Mean [SD]
Clinical characteristics			
Sex: male	10 (43.5)		
Age (years)			44.2 [± 9.78]
Symptom duration (years)			7.7 [± 8.5]
Axial involvement	12(52.2)		
Tobacco use	16 (70)		
Treatment			
NSAIDs	18 (78.3%)		
TNF blockers*	4 (17.4%)		
Biological results			
Sedimentation rate (mm at the first hour)		14	23.3 [± 22.6]
CRP (mg/mL)		8	14.4 [± 20.6]
ASAS criteria			
HLA-B27	7 (30.4)		
Arthritis	9 (39.1)		
Enthesitis	8 (38.8)		
Uveitis	2 (8.7)		
Dactylitis	2 (8.7)		
Psoriasis	6 (26.1)		
Inflammatory bowel disease	3 (13.0)		
Familial history	4 (17.4)		
Inflammatory back pain	22 (95.7)		
Good response to NSAIDs	11 (47.8)		
Biological inflammation (CRP > 5 mg/L)	12 (52.2)		
Clinical evaluation scores**			
BASFI		61.5	59.3 [± 23.0]
BASDAI		5.88	5.45 [± 2.57]
BASMI		3	2.9 [± 2.0]
ASDAS		3	3.3 [± 0.9]

*Anti-TNF: stopped 3 months before the MRI

**BASDAI Bath Ankylosing Spondylitis Disease Activity Index, *BASFI* Bath Ankylosing Spondylitis Activity Functional Index, *BASMI* Bath Ankylosing Metrology Index, *ASDAS* Ankylosing Spondylitis Disease Activity Score

Seven patients were classified with structural sacroiliitis on radiography. Among the 23 patients with active SpA, 20 (87%) presented a BASDAI ≥ 4, and the three other patients were undergoing NSAID treatment failure or had a contraindication for NSAIDs. Moreover, 52.2% of the patients presented with biological inflammation. After the study, 18 patients (78.3%) benefitted from tumor necrosis factor (TNF) blocker therapy.

### Imaging analysis (Table 2)

On CT scans, ten cases of structural sacroiliitis were diagnosed with erosions present on eight exams (mean score of 40.7 ± 21.8) and ankylosis on six exams (mean score of 26.0 ± 18.8), five of which had both lesions. The inter-reader reliability was excellent for the diagnosis of sacroiliitis (CKM[IC95] = 0.82 [0.73; 0.91]) and the scoring of ankylosis (ICC[IC95] = 0.81[0.66; 0.90]) and good for the scoring of erosions (ICC[IC95] = 0.64[0.42; 0.81]).

**Table 2:** Main clinical and imaging results concerning the SpA population

Patient	Age	HLA B27	CRP (mg/L)	BASDAI	Radiographic sacroiliitis	PET positive	PET activity score	PET SUVmax SIJ/sacrum	CT scan structural sacroiliitis	CT scan score for erosions	CT scan score for ankylosis	MRI inflammatory sacroiliits (ASAS criteria)	MRI score for inflammation (SPARCC)
1	52	0	2.2	6.61	0	1	4.0	1.13	0	0	0	0	0
2	39	1	0.5	5.54	0	1	26.3	2.04	1	74.0	25.5	0	0
3	46	0	1.3	7.88	0	0	0	1.48	1	22.5	3	0	0
4	40	0	5	6.69	0	1	17.7	2.61	0	0	0	1	16.3
5	43	0	8.5	7.06	0	1	17.0	1.24	0	0	0	0	0
6	46	1	22.6	8.04	0	1	3.5	1.43	0	0	0	0	1.7
7	59	0	15.6	2.1	1	1	48.0	2.61	1	14.0	0	1	35.7
8	43	1	35.3	4.8	0	1	16.7	1.51	1	0	77.7	0	0
9	41	1	21.5	1.8	1	1	18.0	2.33	1	28.0	16.3	1	17.7
10	30	0	2.6	6.18	0	0	0	1.58	0	0	0	0	0
11	35	0	9.9	3.79	1	1	28.0	1.99	1	10.0	0	0	12.3
12	30	1	29.9	1.3	1	1	15.3	1.45	1	70.7	0	1	9.7
13	34	0	1	6.15	0	1	15.0	1.33	0	0	0	1	12.7
14	45	0	0.6	6.49	0	1	11.3	1.72	0	0	0	1	8.0
15	36	1	3	5.96	1	1	21.7	1.63	1	51.0	11.5	1	9.3
16	51	1	3.9	8.48	0	1	7.7	1.79	0	0	0	0	0
17	28	0	8	8.73	0	1	3.0	1.33	0	0	0	0	3.7
18	66	0	93.5	8.2	1	1	14.3	2.18	1	26.3	22	0	0
19	55	0	9	6.08	0	0	0	1.28	0	0	0	0	0
20	55	0	0.2	9.12	0	1	24.0	1.88	0	0	0	0	0
21	50	0	1.1	5.94	0	1	6.3	1.39	0	0	0	1	16.7
22	51	0	30.2	5.13	0	1	36.3	1.66	0	0	0	1	12.3
23	42	0	26	5.93	1	1	30.7	2.31	1	69.7	0	1	49

*BASDAI* Bath Ankylosing Spondylitis Disease Activity Index, *SUVmax SIJ/sacrum* ratio between the SUVmax for each SIJ and the SUVmax in the center of the sacrum, *SPARCC* SpondyloArthritis Research Consortium of Canada

On MRI, ten cases of inflammatory sacroiliitis were recognized with a mean inflammation score (SPARCC) of 18.7 ± 9.4. The inter-reader reliability was good for the diagnosis of sacroiliitis (ICC[IC95] = 0.53[0.41; 0.64]) and excellent for the scoring of inflammation (ICC[IC95] = 0.95[0.90; 0.98]).

Twenty ^18^F-NaF PET/CT exams were positive with a mean activity score of 18.2 ± 8.7. The mean SUVmax SIJ/sacrum was 1.78 ± 0.35 for positive exams and 1.45 ± 0.66 for negative exams. The inter-reader reliability was low for the diagnosis of sacroiliitis (CKM[IC95] = 0.29[0.18; 0.40]) and good for the activity score (ICC[IC95] = 0.56[0.32; 0.76]). For the SUVmax SIJ/sacrum ratio, the inter-reader reliability was good for the right SIJ (ICC[IC95] = 0.70[0.41; 0.86]) and excellent for the left SIJ (ICC[IC95] = 0.90[0.78; 0.96]).

### Comparison between ^18^F-NaF PET/CT and CT scan (Fig. 2)

On a binary approach, there was no significant correlation between a positive PET and structural sacroiliitis on CT scan (*p* = 1). Among the 20 positive PET cases, there were 9 positive and 11 negative CT scans; 3 PET scans were negative, 1 with a positive CT scan and 2 others with negative CT scans.

**Fig. 2:**
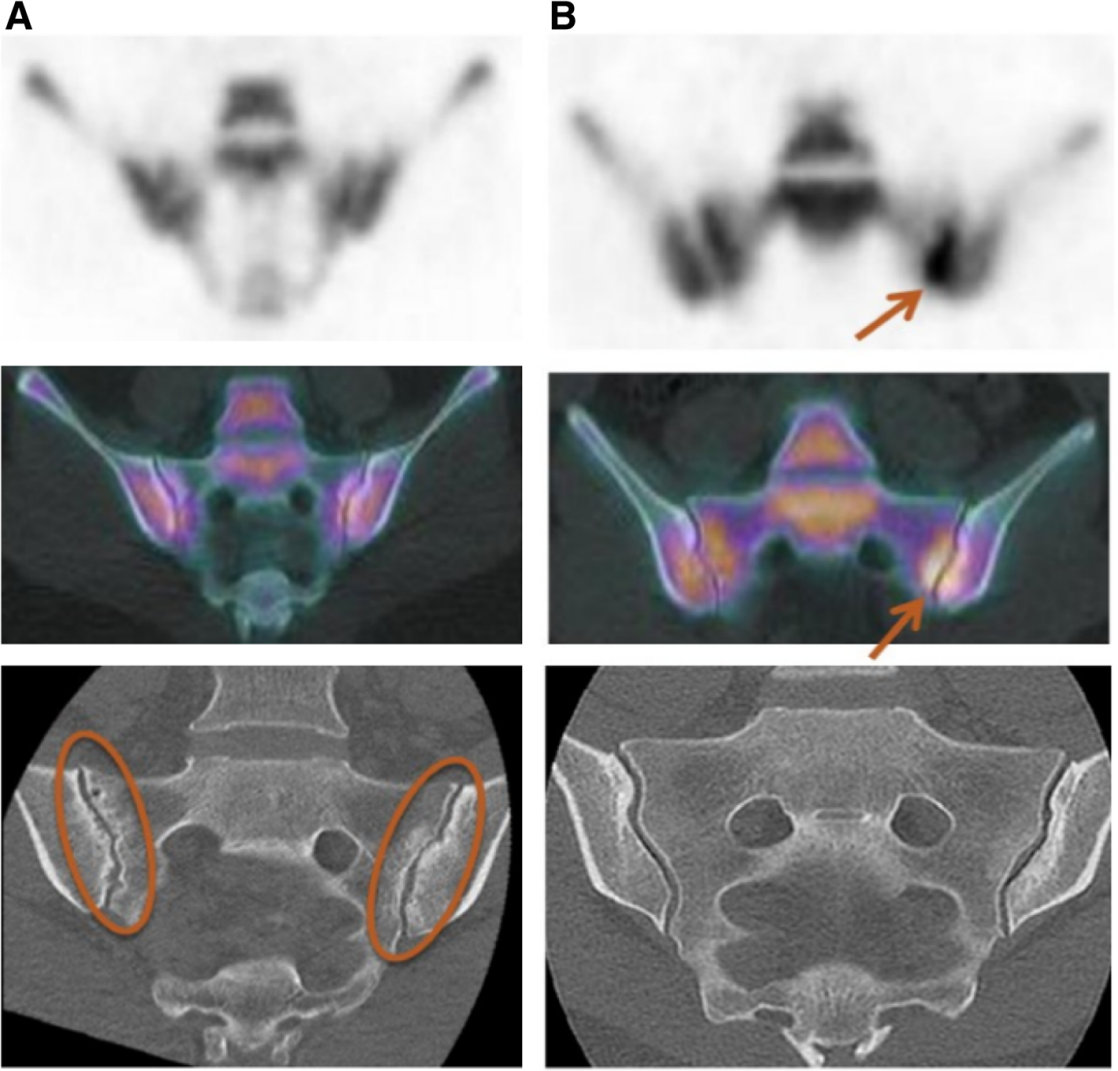
Two examples of discordance between PET/CT and CT scan. A negative PET/CT with structural sacroiliitis on CT scan and multiple erosions (**a**), and a positive PET/CT without structural lesions (erosion or ankylosis) on CT scan (**b**)

For quantitative assessments, there was also no significant correlation between the PET activity score and erosion scores (ICC[IC95] = 0.30[− 0.13; 0.63]; *p* = 0.2) and ankylosis (ICC[IC95] = 0.07[− 0.35; 0.47]; *p* = 0.7). Similarly, the SUVmax SIJ/sacrum ratio was not correlated with erosion scores (ICC[IC95] = 0.25[− 0.18; 0.60]; *p* = 0.2) or ankylosis (ICC[IC95] = − 0.02[− 0.43; 0.40]; *p* = 0.9).

### Comparison between ^18^F-NaF PET/CT and MRI (Fig. 3)

There was no significant correlation between a positive PET and inflammatory sacroiliitis on MRI on a binary approach (*p* = 0.2). All three negative PET scans were also negative on MRI, but only ten of the positive PET scans were positive on MRI, whereas the other ten were negative.

**Fig. 3:**
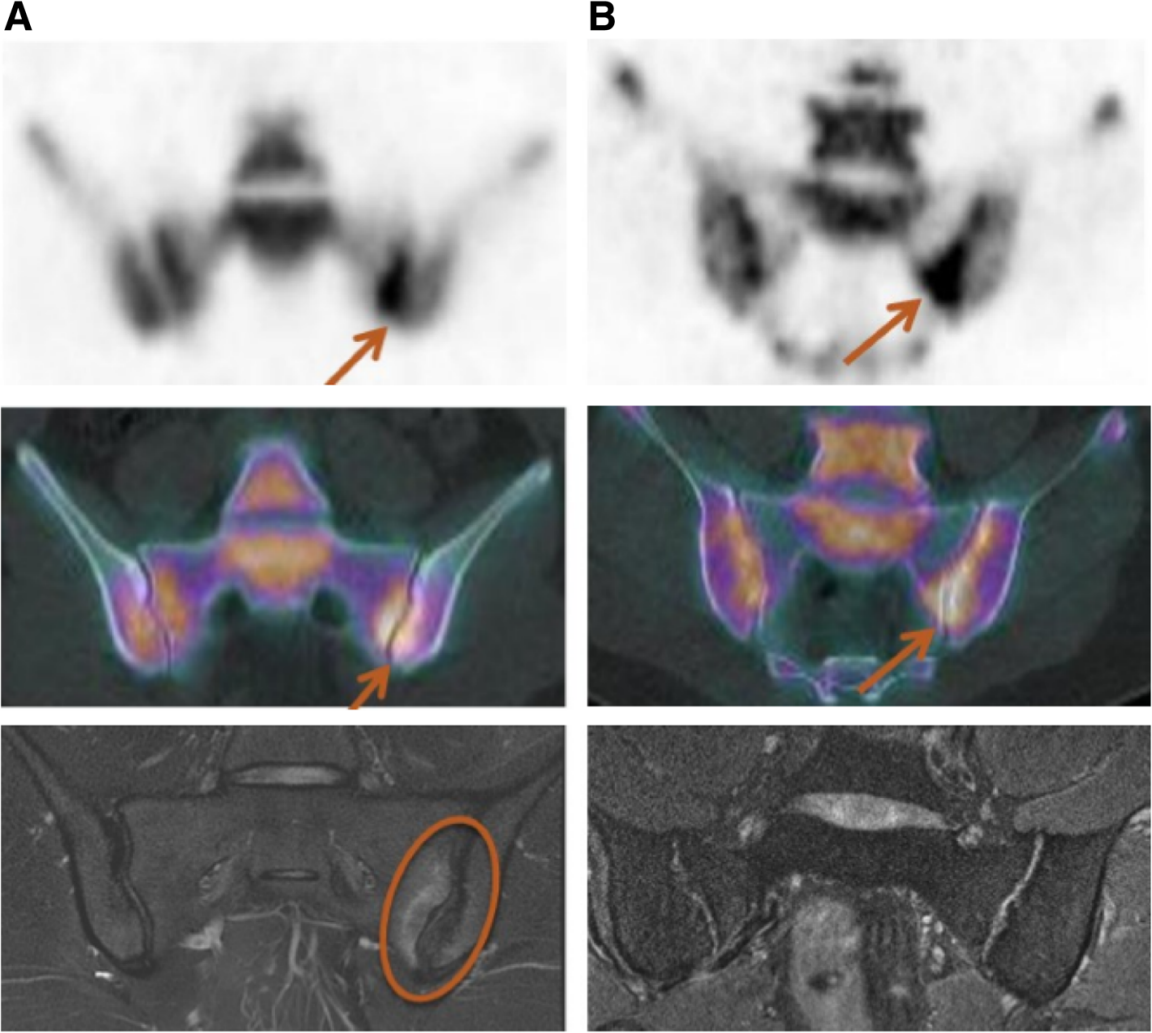
Concordance (**a**) and discordance (**b**) between a positive PET and inflammatory sacroiliitis

For quantitative assessments, there was a significant correlation between the PET activity score and the inflammation score (ICC[IC95] = 0.61[0.26; 0.82]; *p* = 0.001) and between the SUVmax SIJ/sacrum ratio and the inflammation score (CC[IC95] = 0.56[0.19; 0.79]; *p* = 0.004).

### Comparison between ^18^F-NaF PET/CT and clinical and biological parameters

The PET activity score was only correlated inversely with BASDAI (CC[IC95] = − 0.44[− 0.72; − 0.04]; *p* = 0.03) and BASFI (CC[IC95] = − 0.48[− 0.74; − 0.08]; *p* = 0.02), but not with BASMI (*p* = 0.5), ASDAS (*p* = 0.9), and biological inflammation (*p* = 0.5).

The SUVmax SIJ/sacrum ratio was not correlated with any of the clinical or biological parameters.

### ^18^F-NaF PET/CT in radiographic versus non-radiographic SpA (Table 3)

Among the seven patients with radiographic sacroiliitis, all of them presented with structural sacroiliitis on CT scan. Five of these patients had inflammatory sacroiliitis on MRI. In this population, the PET activity score was higher than for the patients without radiographic sacroiliitis.

**Table 3:** Comparison between radiographic and non-radiographic SpA

Radiographic sacroiliitis	Positive (*n* = 7)	Negative (*n* = 16)
^18^F-NaF PET/CT		
Positive (*n*)	7 (100%)	13 (81.2%)
PET activity score (mean)	25.1 ± 8.9	11.8 ± 8.8
SUVmax SIJ/S (mean)	2.1 ± 1.1	1.6 ± 0.4
CT scans		
Structural sacroiliitis (*n*)	7 (100%)	3 (19%)
Erosion score (mean)	37.1 ± 22.8	6.0 ± 10.6
Ankylosis score (mean)	7.1 ± 8.1	6.6 ± 11.2
MRI		
Inflammatory sacroiliitis (*n*)	5 (71.4%)	5 (31.3%)
Inflammation score (mean)	19.1 ± 13.3	4.5 ± 5.5

Sixteen patients did not have radiographic sacroiliitis; three of these patients had structural sacroiliitis on CT scan, and 13 had a positive PET scan. The PET activity score and the mean inflammation score were lower than for patients with non-radiographic SpA.

## Discussion

This study evaluating the performance of ^18^F-NaF PET/CT for the diagnosis of sacroiliitis in a population of axial SpA showed that there were twice as many positive PET scans as there were MRI and CT scans. This finding suggests that ^18^F-NaF PET may be more sensitive than MRI or CT scans for the detection of inflammatory and/or structural sacroiliitis by detecting early or scar lesions not visible on CT scans or MRI.

To our knowledge, this study is the first to compare ^18^F-NaF PET and CT scan for the diagnosis of structural sacroiliitis. In our study, there was no correlation between these two imaging techniques. Buchbender and colleagues already demonstrated the absence of a correlation between the ^18^F-NaF PET uptake and the presence of erosions, ankylosis, or sclerosis but only on MRI [21]. These results reinforce the idea that PET imaging can detect early bone remodeling processes before erosions or ankylosis is present.

Furthermore, we found a significant correlation between both the PET activity score and the SUVmax SIJ/sacrum ratio and the inflammation score on MRI. The concordance between ^18^F-NaF PET and MRI has also already been evaluated in different studies. Buchbender and colleagues [21] used hybrid ^18^F-Fluoride PET/MRI to show that bone marrow edema (BME) detected on MRI is associated with osteoblastic activity, while the combination of BME and fat deposition showed the highest ^18^F-F uptake. Fischer and colleagues [19] found that the correlation between the uptake detected on ^18^F PET and the inflammation on MRI was moderate for the SIJ (kappa = 0.64) and poor for the spine (kappa = 0.25). To explain these results, they suggested that ^18^F PET/CT might be able to detect the anabolic repair process leading to new bone formation regardless of the definite pathophysiological pathway.

In this study, we found high uptake values of ^18^F-NaF on SIJ in patients with axial SpA, without correlation with CT scan lesions. These results imply that ^18^F-NaF PET might detect an early bone remodeling process before structural lesions are visible on CT scan, suggesting the presence of an osteoid tissue not yet mineralized. Surprisingly, when erosions and ankylosis are present, this osteoid tissue seems to be minimal or absent, as it is not detected by ^18^F-NaF PET. However, we found a significant correlation between ^18^F-NaF PET and inflammation on MRI, even though it is observed in only half of the patients. According to these results, two pathophysiological pathways may be involved to explain the ossification process: one initiated by prior inflammation, and a second one without presence of inflammation on MRI.

In a recent study, biopsy procedures were used to collect material from PET-positive lesions in the spine for immunohistochemistry. These PET-positive lesions corresponded to osteoid formation and osteoclasts along with cell infiltrates in areas with both bone and connective tissue, which were largely absent in PET-negative lesions [22]. This study also showed that ^18^F-NaF PET/CT may detect changes in bone formation in AS during treatment, as one third of the PET-positive lesions in the spine disappeared after 12 weeks of TNF blocker treatment in their population of 12 patients with AS. In our study, patients continued their NSAID treatment but none of them were under biologic treatment for at least 3 months. Jarrett et al. demonstrated that NSAIDs have small influence on inflammatory lesions on MRI [23], but it still has to be evaluated on ^18^F-NaF PET.

The main strength of our study lies in its strong methodology. In fact, we based our results on a lecture by three different readers for CT scans, MRI, and ^18^F-NaF PET/CT. In other studies evaluating the performance of PET, the analysis of the imaging techniques was made by only one or two readers. We found that the inter-reader correlation for the qualitative assessment in PET was mediocre, which may be explained by the subjective nature of this evaluation on a complex joint with multiple bone superpositions. This issue suggests that a validation by three readers, with an agreement of at least two readers, seems to be more appropriate.

Moreover, in previous studies, the PET analysis was based only on a qualitative analysis [16, 19, 23] and/or on the SUVmax [18, 21], but an activity score has never been used, which may permit a better evaluation of the SIJ. Indeed, the inter-reader correlation was higher with a quantitative assessment when compared with a qualitative one, which justifies its utilization for a more accurate evaluation of the SIJ by PET imaging. In our study, we also calculated the ratio between the SUVmax for each SIJ and the SUVmax in the center of the sacrum, which makes it less dependent on the examination conditions, including the injected dose of radiotracer, but also on the individual uptake characteristics. The use of SUVpeak as a measuring tool instead of SUVmax could be relevant because it is less sensitive to noise. However, other studies on ^18^F-NaF PET/CT were based only on SUVmax, which is why we chose this assessment, since it permitted us to compare our results to those of the literature.

In our study, 7 AS and 16 non-radiographic SpA patients were included, which is different than what has been reported in the literature. In fact, in most of the studies, the evaluation of PET imaging is conducted on a population of AS [16, 18, 19, 21], but we found that PET imaging can be positive for non-radiographic sacroiliitis. In a study by Toussirot et al., no uptake of ^18^F-NaF PET was noted in SpA without structural sacroiliitis on radiography and also without inflammatory sacroiliitis on MRI [24]. Subgroup analysis between radiographic and non-radiographic SpA should be evaluated on a larger population in order to confirm these results.

We also found a significant correlation between the PET activity score and clinical activity scores (BASDAI and BASFI) but not with inflammation and structural changes, which is consistent with the results in the literature [23, 25, 26]. However, this outcome has to be taken with caution because it was not confirmed by the SUVmax and SUVmax SIJ/sacrum ratio. Moreover, spine assessment was not performed, limiting any comparison with clinical and biological parameters.

The performance of ^18^F-NaF PET/CT for the diagnosis of sacroiliitis has already been evaluated by Strobel and colleagues [18] in a population of 15 patients with active AS in comparison with 13 patients with non-traumatic mechanical low back pain for at least 3 months. In that study, they found a sensitivity, specificity, and accuracy of 80%, 77%, and 79%, respectively, for the diagnosis of sacroliitis, with a mean SUV SIJ/sacrum ratio of > 1.3 as a cut-off value, in comparison with radiography. Furthermore, it appeared that the sensitivity of PET imaging was better for grade 3 sacroiliitis (94%), which suggests that increased uptake might be correlated with post-inflammatory repair associated with osteoproliferation. In that study, as in ours, structural damages relative to osteoarthritic lesions such as osteophytes or intraarticular gas were not taken into consideration.

The main limitation of our study is the small number of examined patients, which requires the statistical analyses to be interpreted with caution. The quantitative approach, which had never been done before, aimed to compensate for this limitation.

Another limitation is the absence of a control group to assess the sensibility and specificity of ^18^F-NaF PET/CT for the diagnosis of sacroiliitis. Indeed, we did not consider the presence of structural damages caused by osteoarthritis which may be detected by ^18^F-NaF and may be the source of false positivity. Likewise, the possibility of overuse of SIJ or anatomical abnormalities such as accessory sacroiliac joint or lumbosacral transitional vertebrae, which can cause inflammation on MRI, was not evaluated. The impact of these modifications on ^18^F-NaF PET uptake could be evaluated in another study with a control group.

Also, all examinations were performed within a month, which is a short term, but does not formally avoid the potential risk of changes between exams in a same patient, even if we know by Jarrett et al. [23] that inflammation on MRI is quite stable at 6 months in SpA patients under conventional treatment.

In order to complete these results, a similar study could be conducted on the spine, evaluating the ^18^F-NaF PET/CT uptake, the bone marrow edema on vertebral corners on MRI, and the presence of erosions, syndesmophytes, and vertebral ankylosis on CT scans or X-rays.

## Conclusion

Abnormal uptake of the SIJ on ^18^F-NaF PET is more frequent than inflammatory sacroiliitis on MRI (43.5%) and structural sacroiliitis on CT scan (43.5%) in a population of SpA patients. The comparison among ^18^F-NaF PET/CT, CT scans, and MRI found only a significant correlation between PET-positive lesions and inflammation assessed quantitatively but not with structural damages assessed on binary and quantitative approaches. Further studies with a control group and a larger sample are needed to evaluate the sensitivity and specificity of PET imaging.

## Abbreviations

^18^F-NaF: ^18^F-Sodium fluoride
AS: Ankylosing spondylitis
ASAS: Assessment of SpondyloArthritis international Society
ASDAS: Ankylosing Spondylitis Disease Activity Score
BASDAI: Bath Ankylosing Spondylitis Disease Activity Index
BASFI: Bath Ankylosing Spondylitis Functional Index
BASMI: Bath Ankylosing Spondylitis Metrology Index
CRP: C-reactive protein
CT: Computed tomography
ESR: Erythrocyte sedimentation rate
MRI: Magnetic resonance imaging
NSAIDs: Non-steroidal anti-inflammatory drugs
PET: Positron emission tomography
SIJ: Sacro-iliac joint
SpA: Spondyloarthritis
SPARCC: Spondyloarthritis Research Consortium of Canada
SUV: Standardized uptake value
SUVmax: Maximum standardized uptake value
TNF: Tumor necrosis factor
